# Genetic heterogeneity in Cornelia de Lange syndrome (CdLS) and CdLS-like phenotypes with observed and predicted levels of mosaicism

**DOI:** 10.1136/jmedgenet-2014-102573

**Published:** 2014-08-14

**Authors:** Morad Ansari, Gemma Poke, Quentin Ferry, Kathleen Williamson, Roland Aldridge, Alison M Meynert, Hemant Bengani, Cheng Yee Chan, Hülya Kayserili, Şahin Avci, Raoul C M Hennekam, Anne K Lampe, Egbert Redeker, Tessa Homfray, Alison Ross, Marie Falkenberg Smeland, Sahar Mansour, Michael J Parker, Jacqueline A Cook, Miranda Splitt, Richard B Fisher, Alan Fryer, Alex C Magee, Andrew Wilkie, Angela Barnicoat, Angela F Brady, Nicola S Cooper, Catherine Mercer, Charu Deshpande, Christopher P Bennett, Daniela T Pilz, Deborah Ruddy, Deirdre Cilliers, Diana S Johnson, Dragana Josifova, Elisabeth Rosser, Elizabeth M Thompson, Emma Wakeling, Esther Kinning, Fiona Stewart, Frances Flinter, Katta M Girisha, Helen Cox, Helen V Firth, Helen Kingston, Jamie S Wee, Jane A Hurst, Jill Clayton-Smith, John Tolmie, Julie Vogt, Katrina Tatton–Brown, Kate Chandler, Katrina Prescott, Louise Wilson, Mahdiyeh Behnam, Meriel McEntagart, Rosemarie Davidson, Sally-Ann Lynch, Sanjay Sisodiya, Sarju G Mehta, Shane A McKee, Shehla Mohammed, Simon Holden, Soo-Mi Park, Susan E Holder, Victoria Harrison, Vivienne McConnell, Wayne K Lam, Andrew J Green, Dian Donnai, Maria Bitner-Glindzicz, Deirdre E Donnelly, Christoffer Nellåker, Martin S Taylor, David R FitzPatrick

**Affiliations:** 1 MRC Human Genetics Unit, MRC Institute of Genetics and Molecular Medicine, University of Edinburgh, Edinburgh,UK; 2 Visual Geometry Group, Department of Engineering Science, University of Oxford, Oxford, UK; 3 Medical Research Council Functional Genomics Unit, Department of Physiology, Anatomy and Genetics, University of Oxford, Oxford, UK; 4 Medical Genetics Department, Istanbul Medical Faculty, Istanbul University, Istanbul, Turkey; 5 Department of Clinical Genetics, Academic Medical Center, University of Amsterdam, Amsterdam, The Netherlands; 6 South East of Scotland Clinical Genetic Service, Molecular Medicine Centre, Western General Hospital, Edinburgh, UK; 7 Medical Genetics Unit, St George's University of London, London, UK; 8 North of Scotland Regional Genetics Service, Clinical Genetics Centre, Aberdeen,UK; 9 Department of Medical Genetics, University Hospital of Northern Norway, Tromsø,Norway; 10 Sheffield Children's Hospital, NHS Foundation Trust, Sheffield, UK; 11 Northern Genetics Service, Newcastle upon Tyne Hospitals, Newcastle upon Tyne, UK; 12 Department of Clinical Genetics, Alder Hay Children's Hospital, Liverpool, UK; 13 Northern Ireland Regional Genetics Service (NIRGS), Belfast City Hospital, Belfast, UK; 14 Weatherall Institute of Molecular Medicine, John Radcliffe Hospital, University of Oxford, Oxford,UK; 15 Clinical Genetics Department, Great Ormond Street Hospital, London, UK; 16 North West Thames Regional Genetics Service,Kennedy-Galton Centre, North West London Hospitals NHS Trust, Harrow, UK; 17 West Midlands Regional Clinical Genetics Service,Birmingham Women's Hospital, West Midlands,UK; 18 Wessex Clinical Genetics Service, Princess Anne Hospital, Southampton, UK; 19 Department of Genetics, Guy's Hospital, Guy's and St Thomas’ NHS Foundation Trust, London, UK; 20 Clinical Genetics, Yorkshire Regional Genetics Service, Leeds,UK; 21 Institute of Medical Genetics, University Hospital of Wales, Cardiff, UK; 22 Department of Clinical Genetics, The Churchill Hospital Old Road, Oxford, UK; 23 SA Clinical Genetics Service, Women's & Children's Hospital, Adelaide,Australia; 24 Department of Paediatrics, University of Adelaide, Adelaide, Australia; 25 West of Scotland Regional Genetics Service, Ferguson-Smith Centre for Clinical Genetics, Yorkhill Hospital, Glasgow, UK; 26 Department of Medical Genetics, Kasturba Medical College, Manipal University, Manipal, India; 27 Department of Medical Genetics, Cambridge University Addenbrooke's Hospital, Cambridge,UK; 28 Faculty of Medical and Human Sciences, Manchester Centre for Genomic Medicine, Institute of Human Development, University of Manchester, Manchester Academic Health Science Centre (MAHSC), Manchester, UK; 29 Department of Dermatology, Kingston Hospital NHS Trust, Surrey, UK; 30 Medical Genetics Laboratory of Genome, Isfahan University of Medical Sciences, Isfahan, Iran; 31 National Centre for Medical Genetics, Our Lady's Children's Hospital, Dublin 12, Ireland; 32 Department of Clinical and Experimental Epilepsy, UCL Institute of Neurology, London, UK; 33 School of Medicine and Medical Science, University College Dublin, Dublin 4, Ireland; 34 Genetics and Genomic Medicine Programme, UCL Institute of Child Health, London, UK

**Keywords:** Molecular genetics, Copy-number, Clinical genetics

## Abstract

**Background:**

Cornelia de Lange syndrome (CdLS) is a multisystem disorder with distinctive facial appearance, intellectual disability and growth failure as prominent features. Most individuals with typical CdLS have de novo heterozygous loss-of-function mutations in *NIPBL* with mosaic individuals representing a significant proportion. Mutations in other cohesin components, *SMC1A*, *SMC3*, *HDAC8* and *RAD21* cause less typical CdLS.

**Methods:**

We screened 163 affected individuals for coding region mutations in the known genes, 90 for genomic rearrangements, 19 for deep intronic variants in *NIPBL* and 5 had whole-exome sequencing.

**Results:**

Pathogenic mutations [including mosaic changes] were identified in: *NIPBL* 46 [3] (28.2%); *SMC1A* 5 [1] (3.1%); *SMC3* 5 [1] (3.1%); *HDAC8* 6 [0] (3.6%) and *RAD21* 1 [0] (0.6%). One individual had a de novo 1.3 Mb deletion of 1p36.3. Another had a 520 kb duplication of 12q13.13 encompassing *ESPL1,* encoding separase, an enzyme that cleaves the cohesin ring. Three de novo mutations were identified in *ANKRD11* demonstrating a phenotypic overlap with KBG syndrome. To estimate the number of undetected mosaic cases we used recursive partitioning to identify discriminating features in the *NIPBL-*positive subgroup. Filtering of the mutation-negative group on these features classified at least 18% as ‘*NIPBL-*like’. A computer composition of the average face of this *NIPBL*-like subgroup was also more typical in appearance than that of all others in the mutation-negative group supporting the existence of undetected mosaic cases.

**Conclusions:**

Future diagnostic testing in ‘mutation-negative’ CdLS thus merits deeper sequencing of multiple DNA samples derived from different tissues.

## Introduction

Cornelia de Lange syndrome (CdLS, MIM #122470) is a multisystem disorder characterised by intellectual disability, prenatal-onset growth retardation, limb malformations, multiple chronic medical problems and distinctive facial features including low anterior hairline, arched eyebrows, synophrys and long philtrum.^1^ Heterozygous loss-of-function (LOF) mutations in *NIPBL* can be identified in more than half the individuals diagnosed with typical (aka ‘classical’) CdLS.^2–4^ Mosaic mutations in *NIPBL* have recently been reported in 23% of ‘mutation-negative’ cases.^5^ ^6^ Heterozygous mutations in the autosomal genes *SMC3*^7^ and *RAD21*^8^ and heterozygous or hemizygous mutations in the X-linked genes *SMC1A*^7^ ^9^ ^10^ and *HDAC8*^11^ ^12^ have been reported in a combined total of approximately 6% of CdLS cases.^13^ Individuals with mutations in genes other than *NIPBL* mostly have phenotypes that overlap significantly with ‘classical’ CdLS but which lack the associated major malformations and are often atypical in terms of growth and/or facial appearance.

Large-scale structural genomic rearrangements that disrupt the function of known CdLS genes have been reported as affecting *NIPBL*,^13^ ^14^ *SMC1A*,^15^ *HDAC8*^12^ and *RAD21.*^8^ Rare copy number variants (CNVs) involving 1p36.23–36.22, 7p22.3, 17q24.2–25.3, 19p13.3 and 20q11.2-q12 have also been reported in association with CdLS-like features.^16^

The protein products of the known CdLS genes each function in the cohesin complex.^17^ ^18^ The best-studied cohesin function has been its role in sister chromatid cohesion. NIPBL (Scc2 in yeast, Nipped-B in *Drosophila*) mediates the loading of a multimeric ring structure onto metaphase chromosomes.^19–21^ This ring structure is a complex of SMC1A, SMC3, RAD21 and STAG1/2 (Smc1, Smc3, Scc1 and Scc3 in yeast). The release of sister chromatids at the end of metaphase is mediated by cleavage of RAD21 by separase.^22^ The cohesin complex also has a role in transcriptional regulation in non-dividing cells.^23^ ^24^ Release and retention of the cohesin ring on interphase chromatin is regulated by the acetylation status of SMC3. SMC3 is acetylated by ESCO1 or ESCO2 (LOF mutations in which cause Roberts syndrome (MIM #268300)^25^) and deacetylated by HDAC8.^11^ There is growing evidence that NIPBL may regulate gene expression via cohesion-independent mechanisms.^26^ ^27^ It is not yet clear which cellular functions of NIPBL and the cohesin complex cause the clinical phenotype associated with each locus.

Here, we report the results from a comprehensive mutation analysis in a cohort of typical and atypical CdLS which has detected new alleles for all the known genes and confirmed mosaicism of causative mutations in a significant proportion of cases. We also identified a duplication involving *ESPL1* and demonstrated phenotypic overlap between atypical CdLS and other disorders of chromatin function. We employed two techniques in order to estimate the number of mosaic cases that we may have missed. First, recursive partitioning^28^ ^29^ was used to identify features that distinguish individual loci in the mutation-positive patients. Applying the resulting classification trees to the cases with no mutation identified, suggested that there was a large number of *NIPBL*-like cases in this group. Second, we used a described method for creating an averaged face from a collection of facial photographs. Using this approach, the *NIPBL*-like group's averaged face had a gestalt score that was more similar to that of the averaged face of individuals with *NIPBL* mutations than to that of the remainder of the mutation-negative group. We conclude that undetected mosaicism, at least for mutations in *NIPBL*, is a plausible explanation for mutation negativity in our current screening strategy.

## Methods

### Patient ascertainment

All patients were referred by experienced clinical geneticists or paediatricians to the MRC Human Genetics Unit for research genetic analysis with a diagnosis of CdLS or possible CdLS. Growth data (weight, length/height and occipital frontal circumference (OFC)) were requested at birth and also at the most recent assessment. Each individual was scored using published diagnostic criteria for CdLS.^1^ Severity scores^30^ were calculated for each individual for whom phenotypic data were available. A score of <15 is considered mild, 15–22 moderate and >22 severe.

### Facial gestalt scores

Single anteroposterior (AP) facial photographs of individuals referred for research genetic testing were assigned a value between 1 and 10 (1=highly atypical, 10=highly typical) based on facial gestalt only. This scoring was performed independently by three experienced clinical geneticists (DRF, RCMH, AKL). The scorers were blinded to the genotype information. Since the three observations were highly correlated (Pearson correlation ≥0.60, p≤1×10^−10^), the mean score for each patient was used for further analysis.

### Mutation analysis by Ion AmpliSeq-Ion PGM, and Sanger sequencing

An AmpliSeq panel of 287 amplicons encompassing the coding sequences of *NIPBL*, *SMC1A*, *SMC3*, *HDAC8*, *RAD21* and *ESPL1* was designed using the Ion AmpliSeq Designer tool (http://www.ampliseq.com) (Life Technologies IAD 27407). Library preparation was performed according to manufacturer's instructions. Libraries were barcoded (Life Technologies) and quantified using a Bioanalyzer High Sensitivity assay (Agilent Technologies). Next-generation sequencing was performed on an Ion PGM (Life Technologies).

Sequence alignment and variant calling were carried out using the software NextGENe (Soft Genetics), rejecting reads where ≥2 bp had a quality score of ≤12. Sequence alignment was performed with ≥12 bp matching ≥65% of the reference. Sequence identifiers: *NIPBL*, NC_000005; *SMC1A*, NC_000023; *SMC3*, NC_000010; *HDAC8*, NC_000023; *RAD21*, NC_000008; *ESPL1*, NC_00012; *ANKRD11*, NC_000016.10.

### Deep sequencing of the *NIPBL* genomic locus

DNA probes were designed using the software NimbleDesign (https://design.nimblegen.com) to capture a region of 229 061 bp encompassing the *NIPBL* genomic locus (chr5:36 856 861–37 085 921, hg19). Library preparation and locus-specific capture were performed using the SeqCap EZ Choice Library kit (Roche NimbleGen) and TruSeq dual-index barcodes (Illumina) according to manufacturer's instructions. All captured libraries were combined and paired-end sequenced in a single lane of a HiSeq-2000 instrument (Illumina).

### Library preparation, exome capture and variant calling

Library preparation for exome capture of five CdLS cases was performed using the SureSelect Human All Exons 50 Mb kit (Agilent Technologies) for Illumina paired-end sequencing on a HiSeq 2000 sequencing system. Quality control, sequence alignment and variant calling were performed as described previously.^31^

### Pyrosequencing

Allelic quantification of the mosaic *NIPBL* c.1435C>T mutation in individual II:1 (Family 3061) was carried out by pyrosequencing.^32^ Oligonucleotide primers were designed with PyroMark Assay Design Software V.20 (Qiagen) and are available upon request. Pyrosequencing was carried out on a PyroMark Q24 Vacuum Prep Workstation (Biotage). The allele quantification (AQ) mode of PyroMark Q24 software (Biotage) was used for peak quantification.

### Array-based comparative genomic hybridisation (aCGH)

Analysis of genome-wide DNA copy number was performed using the Nimblegen 135k microarray platform (Roche Nimblegen) as described previously.^33^ Results were compared with the Database of Genomic Variants (http://dgv.tcag.ca/dgv/app/home) and polymorphic CNVs excluded. The deletion of 1p36.33-p36.32 was confirmed using the Nimblegen 720k whole-genome array (median probe spacing of approximately 2.5 kb).

### Genomic copy number assay of *ESPL1*

Genomic copy number analysis of *ESPL1* was carried out using a TaqMan CNV assay (assay ID Hs_02074777_cn) (Applied Biosystems) with a probe located in exon 3 of *ESPL1*. Real-time PCR was carried out on an HT7900 instrument (Applied Biosystems) and CNV analysis was performed using CopyCaller software V.1.0 (Applied Biosystems).

### Recursive partitioning

Classification trees were created and visualised in R using the packages RPART, TREE, RATTLE and RPART.PLOT. RPART was invoked using the parameters, method=‘class’, minsplit=3 and maxdepth=3.

### Construction of average faces

We applied the methodology described in Ferry *et al*^34^ to create average faces of patients. Briefly, each face was annotated with 36 feature points (constellation). Within a group, all constellations were registered to each other (Procrustes algorithm) and averaged to obtain an average shape constellation. A reference face mesh was generated via Delaunay triangulation and the appearance of each individual face was mapped to the average face mesh by piecewise affine warping. Averaging of the mapped appearances gave the average face.

## Results

### Mutations in known CdLS genes

**Figure 1:**
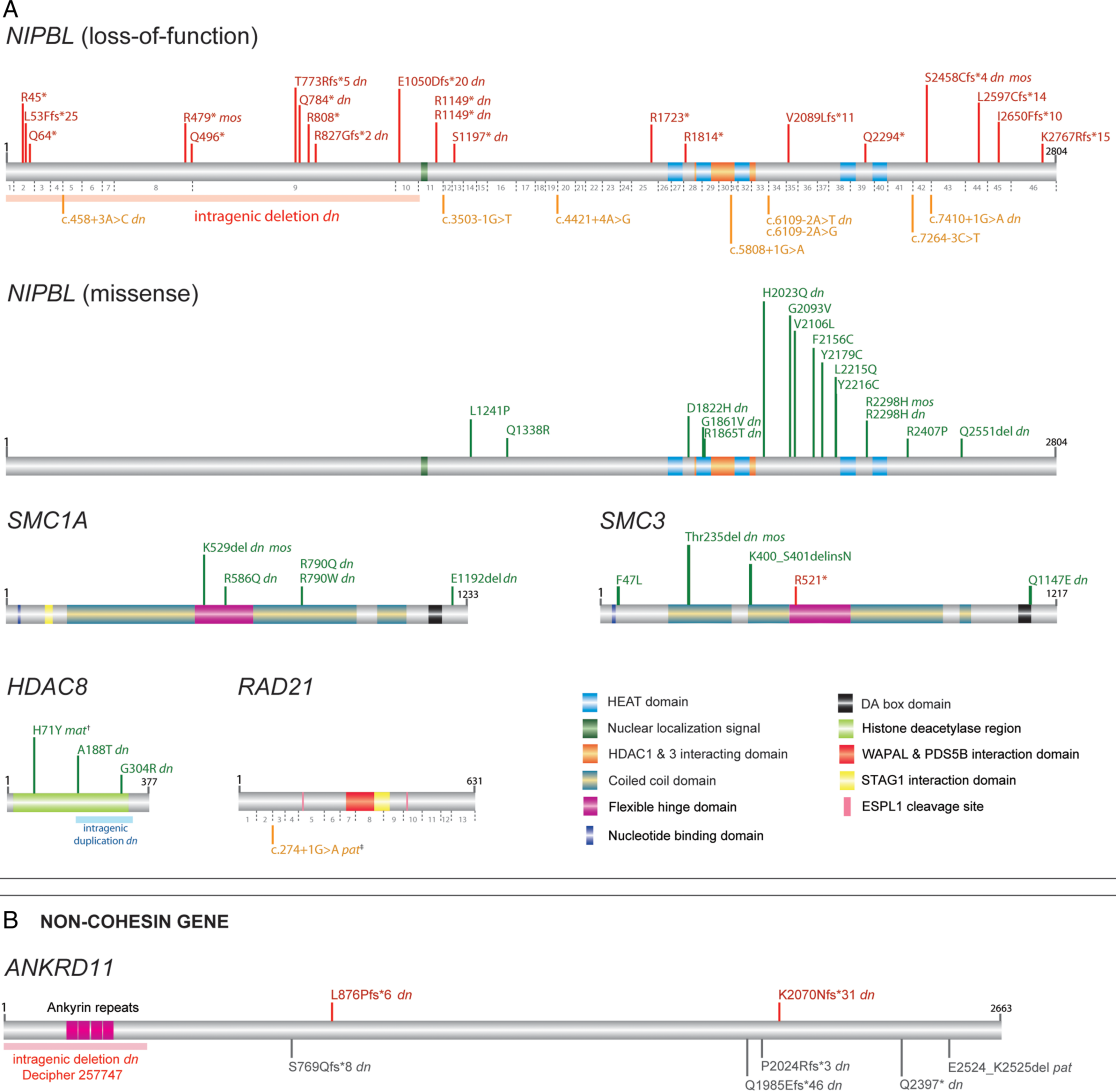
(A) Schematic representation of mutations identified in *NIPBL*, *SMC1A*, *SMC3*, *HDAC8* and *RAD21* within the MRC Human Genetics Unit Cornelia de Lange syndrome cohort. Mutations involving *NIPBL* are divided into two classes of loss-of-function (top panel) and missense (bottom panel) with numbers of *NIPBL* coding exons shown below the NIPBL protein in grey. Position of the *NIPBL* intragenic deletion^43^ is marked by a pink block. The intragenic duplication in *HDAC8*^12^ is marked by a blue block. Positions of all mutations are drawn to scale along the protein product of the longest isoform, and represented in red (loss-of-function), orange (splice-site) or green (missense). †, denotes the *HDAC8* p.(His71Tyr) mutation inherited from a similarly affected mother with skewed X-chromosome inactivation.^12^ ‡, denotes the *RAD21* essential splice-site mutation, inherited from an apparently unaffected father. (B) Two novel frameshift mutations identified by whole-exome (K2070Nfs*31) and Sanger sequencing (L876Pfs*6) are shown in red over the ANKRD11 protein (grey block). The intragenic deletion involving *ANKRD11* is depicted by a pink block (Decipher DDD-EDB257747). *ANKRD11* mutations reported previously^38^ are shown in grey under the ANKRD11 protein block. The first and last amino acid numbers are marked in black; *dn*, confirmed de novo; *mos*, mosaic mutation; *mat*, inherited maternally; *pat*, inherited paternally. Protein accession numbers used are as follows: NIPBL, NP_597677.2; SMC1A, NP_006297.2; SMC3, NP_005436.1; HDAC8, NP_060956.1; RAD21, NP_006256.1; ANKRD11, NP_001243111.1. DDD, Deciphering Developmental Disorders.

Using a custom AmpliSeq panel coupled with Ion PGM sequencing, 163 individuals with CdLS or a CdLS-like disorder were screened. Coverage of the open reading frames of the target genes was as follows; *NIPBL* (98%), *SMC1A* (100%), *SMC3* (92%), *HDAC8* (99%) and *RAD21* (100%). Including four intragenic CNVs detected by array-based comparative genomic hybridisation (aCGH) and one single nucleotide variant detected by exome sequencing, 63 causative mutations were identified in total: *NIPBL*, 46 (24 males:22 females) (28.2%)*; SMC1A*, 5 (1 male:4 females) (3.1%); *SMC3*, 5 (2 males:3 females) (3.1%); *HDAC8*, 6 (all female) (3.6%) and *RAD21*, 1 (female) (0.6%) (figure 1A and see online supplementary table S1). Of the 63 mutations, DNA was available from both parents in 32 families and of these, 30 of the mutations were shown to have occurred de novo. The two inherited variants were a maternally inherited *HDAC8* missense mutation and a paternally inherited *RAD21* essential splice site mutation (figure 1A). The entire *NIPBL* genomic locus was sequenced in 19 mother/father/affected child trios (57 individuals) using a targeted next-generation sequencing approach in order to identify de novo non-coding mutations and deep intronic mutations. Approximately 75% of the 229 kb region was sequenced with a depth of ≥250× (mean coverage 627×) which included the *NIPBL* coding and non-coding exons, its intronic and splicing regions and 20 kb of the upstream promoter region. The results identified no further plausibly pathogenic mutations.

### Mosaicism for mutations in known CdLS genes

**Figure 2:**
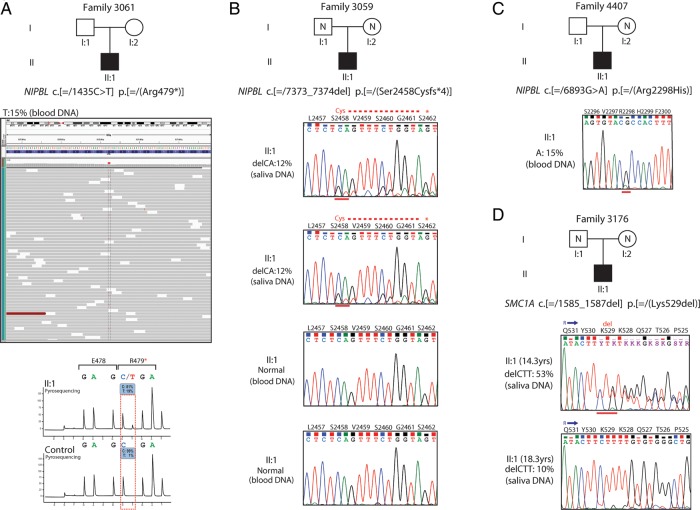
Mosaic mutations identified by next-generation sequence analysis. (A) A mosaic nonsense mutation identified by whole-exome sequencing in *NIPBL* (alternative allele shown in red) in approximately 15% of the reads (grey bars) (individual II:1, Family 3061) (top). The C>T substitution was confirmed by pyrosequencing (bottom) and found to be at similar levels to the exome data (15%–19%) as compared with a control DNA. (B) Sanger sequence confirmation of a de novo 2 bp deletion mutation in *NIPBL* as detected by Ion AmpliSeq-Ion PGM sequencing at 12% in two saliva-derived DNA samples. The mutation appears to be completely absent in two blood-derived DNA samples from the same case (individual II:1, Family 3059). (C) Sanger sequence confirmation of a missense *NIPBL* mutation identified by Ion PGM sequencing at 15% in a blood-derived DNA sample (individual II:1, Family 4407). (D) Sanger sequence confirmation of a de novo in-frame deletion of 3 bp identified by Ion PGM sequencing in *SMC1A* at significantly different levels in two saliva-derived DNA samples: 53% and 10% from the same case (individual II:1, Family 3176) at ages of 14.3 years and 18.3 years, respectively.

Each of the cases discussed in this section are included in the total numbers for mutations in each gene documented in the preceding paragraph. On exome analysis, a mosaic mutation in *NIPBL* coding exon 8 (c.[=/1435C>T] p.[=/(Arg479*]) was identified in a male CdLS case (Family 3061, individual II:1) (figure 2A), who had previously been scored negative following Sanger sequencing of *NIPBL*. This nonsense mutation was found in 15% of the reads (31 out of 206 reads). The C>T conversion was confirmed by pyrosequencing in which the mutated allele was estimated at approximately 19% as compared with a control (figure 2A). In addition, two *NIPBL* mutations (figure 2B, C, see online supplementary figure S1), one *SMC1A* (figure 2D, see online supplementary figure S1) and one *SMC3,* that were identified on the AmpliSeq analysis were shown to be mosaic. A frameshift mutation in *NIPBL* (individual II:1, Family 3059) was detected at 12% in two saliva-derived DNA samples from the proband, but was apparently absent in two blood-derived DNA samples from the same individual (figure 2B, see online supplementary figure S1). A missense mutation in *NIPBL* (individual II:I, Family 4407) was detected at 15% and 38% in the proband's blood-derived and saliva-derived DNA samples, respectively (figure 2C, see online supplementary figure S1). An in-frame deletion in *SMC1A* (individual II:1, Family 3176) was detected at 53% (14.3 years) and 10% (18.3 years) in saliva-derived DNA samples from the proband (figure 2D, see online supplementary figure S1).

### Rare CNVs not encompassing known CdLS loci

**Figure 3:**
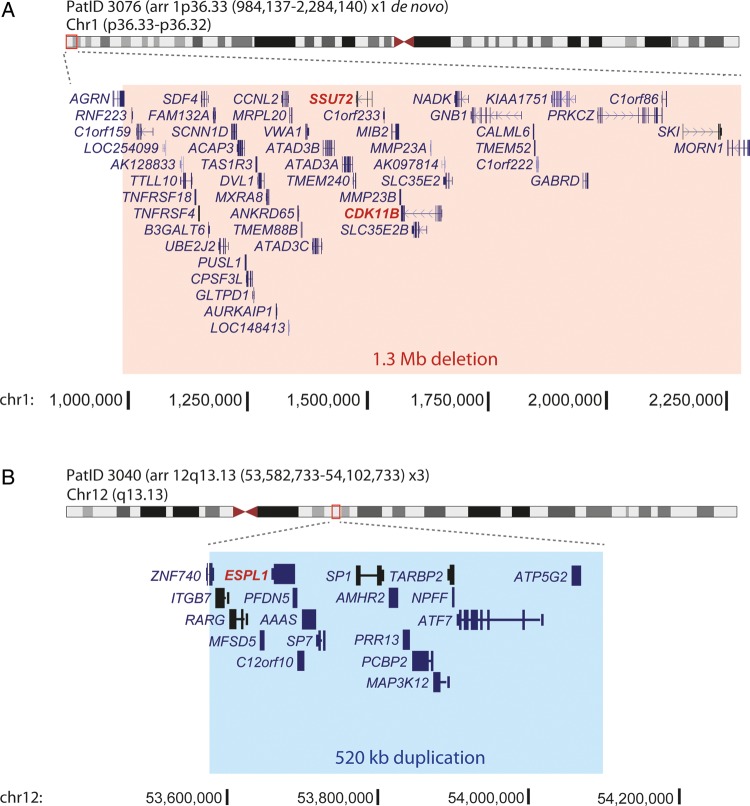
Analysis of genome-wide copy number by array comparative genomic hybridisation. (A) Heterozygous de novo deletion of 1.3 Mb on chromosome 1 (chr1:984 137–2 284 140; hg19) in PatID 3076. (B) Heterozygous duplication of 520 Kb on chromosome 12 (chr12:53 582 733–54 102 733; hg19) in PatID 3040. The regions of deletion and duplication are marked in red and blue boxes, respectively. The genes involved in each chromosomal rearrangement are also shown, with the Cornelia de Lange syndrome candidate genes in each region highlighted in red. The genomic context, marked in black at the bottom of each panel is based on human genome assembly GRCh37/hg19. PatID, patient identification.

aCGH on 90 mutation-negative individuals identified two plausibly pathogenic variants. A de novo heterozygous 1.3 Mb deletion on chromosome 1p36.33 (chr1:984 137–2 284 140; hg19) encompassing 47 genes was detected in a female patient (figure 3A, see online supplementary figure S2A) who presented with a CdLS-like phenotype. A heterozygous 520 kb duplication on chromosome 12q13.13 (chr12:53 582 733–54 102 733; hg19) encompassing 18 genes (figure 3B, see online supplementary figure S2B) was identified in a male patient with a CdLS-like disorder. It was not possible to determine if this duplication had occurred de novo due to unavailability of parental DNA samples. One of the duplicated genes was *ESPL1,* which encodes separase, involved in the release of SMC1A/SMC3 ring from the newly synthesised sister chromatids prior to cell division via cleavage of RAD21. A TaqMan copy number assay was used in order to confirm this duplication (see online supplementary figure S2C) and to screen our patient cohort for further duplications involving *ESPL1*. A total of 80 cases were screened (data not shown). However, no further cases of *ESPL1* copy number gain were identified. The coding sequence of the *ESPL1* gene was also screened in 151 CdLS cases as part of the AmpliSeq panel described above, with the aim of unravelling intragenic mutations in our cohort. However, no non-polymorphic variants were identified. In the course of our study, an intragenic deletion was identified in one of the individuals in our CdLS-like cohort as part of the Deciphering Developmental Disorders (DDD) project^35^ (arr 16q24.3(89 351 798–89 412 086)del: Decipher DDD-EDB257747) representing an intragenic deletion of *ANKRD11* (figure 1B). On review, this boy's phenotype was compatible with a diagnosis of KBG syndrome.

### Whole-exome analysis and targeted resequencing of *ANKRD11*

Whole-exome sequencing was performed on five individuals in whom no mutation had been identified and where genomic DNA of sufficient quantity and quality was available. The mean percentage reads on-target and read depth were 53% and 254×, respectively (see online supplementary table S2). No plausible mutations could be identified in three of the five individuals. As mentioned above, one individual had a mosaic *NIPBL* mutation. Another individual was found to carry a truncating frameshift mutation in coding exon 7 of *ANKRD11* (c.6210_6211del p.(Lys2070Asnfs*31)) which was confirmed as de novo by Sanger sequencing (figure 1B). This girl had an atypical CdLS-like phenotype, but notably had a normal head circumference (OFC=−0.27 SD) as did the boy mentioned above with the intragenic deletion in *ANKRD11*. We, therefore, sequenced the coding and splice regions of *ANKRD11* in 10 further individuals with reported OFC ≥−2.0 SD. Out of the 10 cases analysed, a further de novo case of a 1 bp deletion in *ANKRD11* predicted to result in a truncating frameshift mutation was identified in a female individual (figure 1B). The combined phenotypic data on the three *ANKRD11* cases are shown in online supplementary figures S3, S4 and table S3.

### Genotype-phenotype analysis

**Figure 4:**
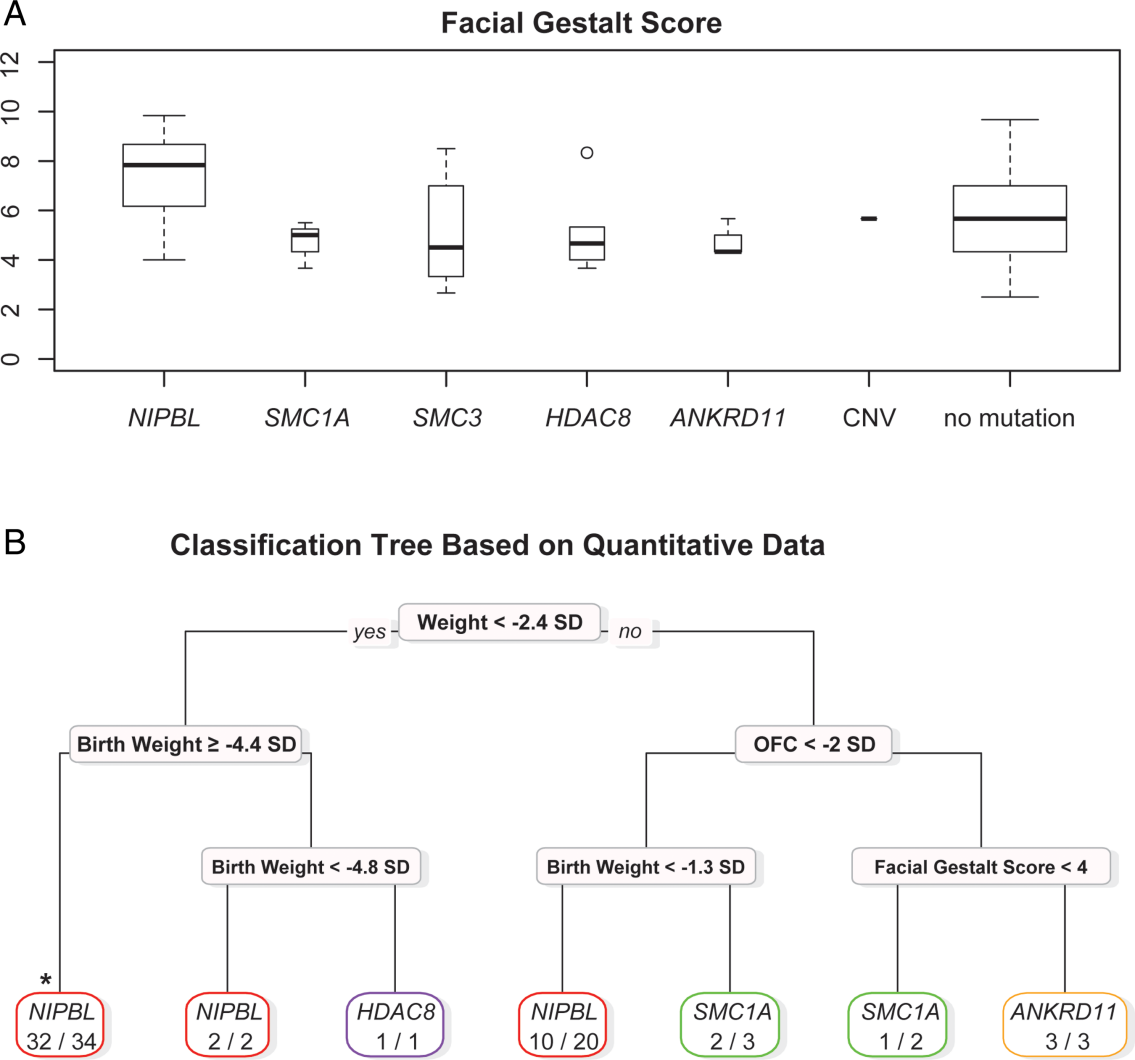
(A) Box plot of the facial gestalt scores for each gene category, genomic rearrangements (copy number variants, CNV) and mutation-negative cases. (B) Classification tree based on prenatal and postnatal growth data, severity and facial gestalt scores and genetic data. *Marks the most *NIPBL*-positive cases classified in a single branch. OFC, occipital frontal circumference.

Quantitative and categorical clinical and developmental information in referred cases was collected in a systematic manner. Few statistically significant differences were observed between the groups if individual components of the phenotype were compared (see online supplementary figure S3 and table S3). For example, the median facial gestalt score was higher in the individuals with *NIPBL* mutations (figure 4A), but the range of scores significantly overlapped with all other groups. The median facial gestalt scores for the individuals with mutations in non-*NIPBL* cohesin-component genes are very similar to those in individuals with *ANKRD11* mutations which have been associated with the ‘non-cohesinopathy’, KBG syndrome. This confirms the general clinical impression that the facial phenotype associated with *SMC1A*, *SMC3* and *HDAC8* are somewhat less discriminative than those associated with *NIPBL*. The pattern of prenatal and postnatal growth highlights some potentially interesting differences, with *NIPBL*, showing progressive growth failure, whereas at least for weight, the *SMC1A* group shows postnatal normalisation (see online supplementary figure S4 and table S3).

### Estimating the number of undetected mosaic mutations

It is clear from the data presented above and those mentioned in the introduction that a significant proportion of individuals with typical CdLS have mosaic mutations. We employed two different approaches in an attempt to estimate the proportion of the mutation-negative cases that may carry undetected mutations by our current mutation analysis protocols: recursive partitioning and the gestalt of computer-averaged faces from subgroups within the cohort.

We used the R package, RPART to define discriminative feature sets within the mutation-positive cases. A simple classification tree using only the prenatal and postnatal growth data and facial gestalt scores is shown in figure 4B. Overall, the *NIPBL* branches classified 56 individuals of whom 44 (78%) had *NIPBL* mutations. The non-*NIPBL* branches held 9 individuals, none of whom had a *NIPBL* mutation. However, one branch of the tree (figure 4B; weight <−2.4 SD, birth weight ≥−4.4 SD), used only two parameters to classify 32 out of 34 (94%) individuals with *NIPBL* mutations. When missing data were removed, all 20/20 remaining cases had *NIPBL* mutations. Applying this strict feature set filter to the mutation-negative group identified 19 out of 103 (18%) individuals with weight <−2.4 SD AND birth weight ≥−4.4 SD. These cases can, therefore, be conservatively classed as *NIPBL*-like.

**Figure 5:**
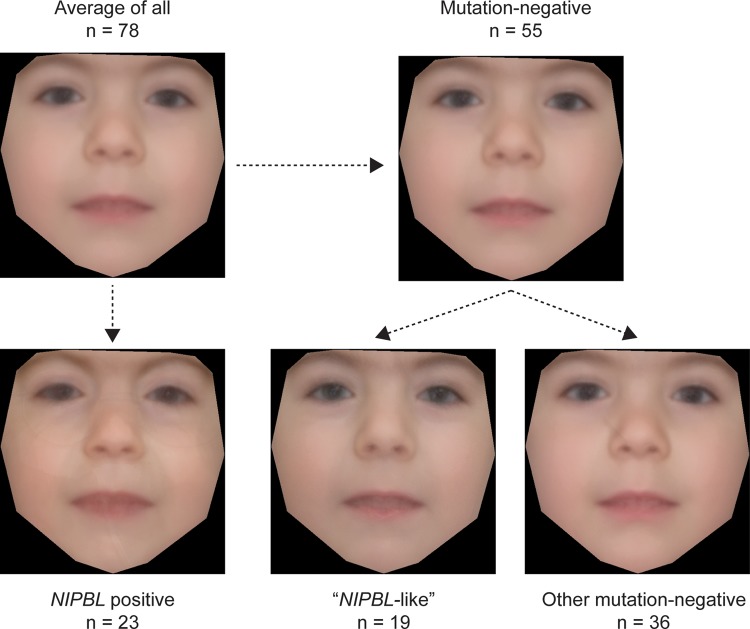
Average faces of affected individuals constructed using average of appearance and shape across patient groups. The average face of the whole cohort (where photographs were available) is shown in the top left hand image. The averaged face of the *NIPBL-*positive subgroup is shown below and that of the subgroup containing all of mutation-negative cases is in the top right image. The *NIPBL*-like and other mutation-negative patient groups are shown in the bottom middle and bottom right image, respectively (n=numbers of individuals that each average face represents).

We used a recently described technique^34^ to create an averaged face for each molecularly defined subset within the cohort (figure 5). All the faces show features typical of CdLS, particularly a long rather featureless philtrum and a thin upper lip. However, the gestalt of the average face for the *NIPBL*-positive group is much more typical of CdLS compared with either that of the whole group or that of the whole mutation-negative group. When the mutation-negative group is split, the *NIPBL*-like subgroup's face also appears more typical than that of other mutation-negative cases, although the difference is less striking.

## Discussion

The combination of massively multiplex PCR technology and next-generation sequencing has revolutionised gene panel-based diagnostic genetic research and clinical analysis. Using these technologies, we screened the coding sequences of the five known causative CdLS genes in 163 individuals with CdLS and CdLS-like phenotypes. As expected, intragenic mutations in *NIPBL* were, by far, the most common identifiable cause. The frequency of intragenic *NIPBL* mutations in our cohort (∼28%) was considerably lower than the ∼50% estimated from published reports.^2^ ^3^ This is almost certainly due to the composition of our cohort, which is deliberately enriched for atypical cases, as we wished to assess the extent of locus and allelic heterogeneity in this group. We used a trio-based pull-down approach to sequence the entire genomic locus of *NIPBL* to identify deep intronic or further mosaic mutations that may be causative, however, none were identified in the 19 families analysed. The frequency of intragenic mutations in *SMC3* in our cohort (3.1%) is considerably higher than previously reported.^7^

3/46 *NIPBL*, 1/5 *SMC1A* and 1/5 *SMC3* mutations in our cohort were mosaic. All three mosaic *NIPBL* mutations were in individuals with severe and typical CdLS, with one of these identified via ultradeep (mean depth >250×) whole-exome sequencing. The mosaic *SMC1A* mutation was an atypical male with moderate growth retardation, whose mutation load in the two saliva samples we had available, varied five-fold (52% and 10%; figure 2). One mosaic *SMC3* mutation was found in a female individual with severe microcephaly and an atypical facial appearance. *HDAC8* mutations have emerged in the last 2 years as a significant contributor to the CdLS-like phenotype. This locus accounted for six (3.6%) cases in our cohort and clinical details of these individuals have been reported elsewhere.^12^ Overall, the pattern of prenatal and postnatal growth in *HDAC8* cases was less severe than in *NIPBL*-positive cases (see online supplementary table S3 and figure S4). We detected no mosaic mutations in *HDAC8*. With regard to the frequency at which we have identified mosaic mutations in this study, the origin of the DNA that we screened may be important; 44% was derived from peripheral blood and 22% from saliva. A recent study identified *NIPBL* mutations in buccal epithelial cells of patients with classical CdLS who had been scored negative by sequencing of blood-derived DNA.^6^ Saliva-derived DNA has several advantages in the analysis of CdLS; first, it is easy to collect and second it is of multitissue origin (bone marrow derived and buccal epithelial cells). Saliva enables multiple samples to be obtained from individuals, and the level of mosaicism and even the presence of the mutation can vary significantly over time (figure 2). The mechanism underlying mosaic mutations in *NIPBL*, *SMC1A* and *SMC3* merits further work, as cases of *NIPBL* mosaicism in our study are indistinguishable clinically from those with a heterozygous LOF mutation. However, selection against mutant cells, particularly in lymphocytes has been suggested as one plausible mechanism.^6^

Of the 90 individuals screened by aCGH, we identified one de novo deletion of 1.3 Mb on chromosome 1p36.33-p36.32 and a duplication of 520 kb on chromosome 12q13.13 encompassing 18 genes (chr12:53 582 733–54 102 733; hg19). Among these, increased dosage of *ESPL1* is a plausible mechanism for cohesin dysfunction and thus, also for CdLS. The endopeptidase ESPL1 (aka separase) is activated via degradation of its inhibitory chaperon, securin, to cleave RAD21 in order to allow the synchronised separation of sister chromatids during the metaphase to anaphase transition.^36^ Overexpression of ESPL1 in mice has been shown to result in the formation of aneuploid tumours in the mammary gland, and ESPL1 is found to be significantly overexpressed in human breast tumours.^37^ We screened 80 other CdLS individuals using a TaqMan CNV assay but did not identify any further cases of *ESPL1* duplication. Finally, we analysed the coding sequences of *ESPL1* in 151 of our CdLS cases as part of the AmpliSeq screen, with the aim of unravelling gain-of-function mutations within this gene. However, no causative mutations were detected.

We performed whole-exome sequencing on five individuals who were mutation-negative to that point. As mentioned above, one individual was mosaic for a nonsense mutation in *NIPBL*. One individual with atypical CdLS had a frameshift mutation in *ANKRD11*, which on testing the parental DNA samples was found to have occurred de novo in the affected child. Coincident with this finding, a de novo intragenic deletion in *ANKRD11* was identified in one of the individuals in our cohort via the DDD project. These two individuals were unusual within our cohort in that they both had head circumferences within the normal range. A screen for intragenic *ANKRD11* mutations in 10 other individuals, with a normal head circumference, revealed one further heterozygous LOF mutation. On review of the facial dysmorphology associated with KBG syndrome,^38^ there is a clear overlap with the features in CdLS. This is borne out by the facial gestalt score in this study, performed blind to the genotype, which suggests that the *ANKRD11* cases are at least as facially similar to classical CdLS as those with mutations in *SMC3* or *HDAC8* (figure 4A). Fortunately, the head growth parameter appears to provide a simple discriminative feature for *ANKRD11*.

In addition to the clinical photographs that we collected to create the gestalt score, we acquired quantitative growth data, developmental milestones and physical characteristics from referred cases using a structured data questionnaire that could be completed either online or by hardcopy. In general, results supported previous studies showing more severe effects on growth and a higher severity score for those with *NIPBL* mutations (and particularly, truncating *NIPBL* mutations).^2^ ^7^ ^9^ ^10^ ^30^ ^39–41^ Limb reduction defects were found in seven individuals with an identified mutation, all of which were in *NIPBL* (see online supplementary table S4). Heart defects (primarily affecting the septum) were found in 32% of individuals with an *NIPBL* mutation, similar to the one-third reported in a previous study.^42^ Two individuals with *HDAC8* mutations also had septal defects, close to the 36% previously reported.^12^ When individual phenotypic characteristics were compared between the genetic groups, few statistically significant differences could be identified. This is probably due to the small numbers in most of the genetic groups and the allelic diversity within *NIPBL*. This latter point is supported by the positive correlation in severity between the mutation classes and growth failure within *NIPBL* (see online supplementary figure S4 and table S3).

One of the most important diagnostic problems in CdLS is how many mutation-negative individuals harbour undetected mosaic mutations in known CdLS genes. In an effort to try to estimate this, we used recursive partitioning to determine if combinations of features could discriminate individual molecular subgroups. Recursive partitioning techniques to create classification trees have been used extensively to aid clinical decision making, particularly in oncology^28^ and cardiovascular disorders.^29^ We created a classification tree that was based only on the quantitative growth data and severity and facial gestalt scores. The tree successfully differentiated the *ANKRD11* cases by head circumference (figure 4B). This tree also enabled us to identify the combination of components which could classify up to a quarter of the individuals in whom no mutation could be found as ‘*NIPBL*-like’. The gestalt of the averaged faces in the subgroups supported the existence of a more typical group within the mutation-negative subgroup. Although this latter analysis is very subjective, CdLS is a diagnosis that is typically made on the basis of facial appearance and most dysmorphologists are very familiar with the gestalt. This is a technique that is potentially broadly applicable as it uses standard facial photographs and could be applied to any syndrome in which there is a characteristic facial appearance. We have not yet done a similar analysis with the other known genes, because the number of facial images is not sufficient and the typical face of the mutation-positive cases is not yet fully appreciated. Statistical approaches to the similarity of averaged faces are being developed and may ultimately replace the human gestalt assessment.^34^ The *NIPBL*-like group are clearly an interesting group on whom to focus future research, as they are likely to be enriched for mosaic cases. Deeper sequencing from a variety of different tissues may be required to determine the optimal diagnostic approach. Our preferred strategy in cases of classical CdLS would involve screening of the five known genes in skin-derived or saliva-derived DNA, aCGH analysis of mutation-negative cases and finally trio-based (proband/mother/father) whole-exome sequencing of the mutation-negative individuals. In cases of atypical CdLS, screening of blood-derived DNA may also be performed.

Another possible explanation for why we were not able to identify causative mutations in the majority of the individuals in our cohort is that the causative mutations lie in other genes. Other genes are yet to be discovered, but the identification of *ANKRD11* mutations in our group suggests that on the basis of facial dysmorphology, at least, there is a significant overlap between cohesinopathies and other chromatin disorders. Our future work will aim to extend the molecular analysis to include trio-based whole-exome approaches and to continue to collect comprehensive clinical and developmental information and facial images from as many cases as possible. It is likely that the differences between the genetic groups will lead to interesting insights into the function of the individual gene products.

## Supplementary Material

Web supplement
