# Supplementary material for: Genetic heterogeneity in Cornelia de Lange syndrome (CdLS) and CdLS-like phenotypes with observed and predicted levels of mosaicism
Source: J Med Genet. 2014 Aug 14;51(10):659–68. doi: 10.1136/jmedgenet-2014-102573 (PMC4173748; doi:10.1136/jmedgenet-2014-102573)
Supplement: Web supplement [file jmedgenet-2014-102573-s1.pdf]

Genetic heterogeneity in Cornelia de Lange syndrome (CdLS) and CdLS-like phenotypes with observed and predicted levels of mosaicism

SUPPLEMENTARY MATERIAL

Supplementary figure S1. Intragenic mosaic mutations identified by Ion AmpliSeq-Ion PGM sequencing. (A) *De novo* mosaic frameshift mutation in *NIPBL* (c.[=/7373_7374del] p.[=/(Ser2458Cysfs*4)] identified in individual II:1 (Family 3059) in 12% of reads in two saliva-derived DNA samples. The sequence data appeared normal in two blood-derived DNA samples from the same case. (B) Mosaic missense mutation in *NIPBL* (c.[=/6893G>A] p.[=/(Arg2298His)]) identified in individual II:1 (Family 4407) at approximately 15% and 38% in blood-derived and saliva-derived DNA samples, respectively. (C) *De novo* mosaic in-frame deletion of 3 bp in *SMC1A* (c.[=/1585_1587del] p.[=/(Lys529del)]) identified at significantly different levels (53% and 10%) in two saliva-derived DNA samples from individual II:1 (Family 3176) collected at ages of 14.3 and 18.3 years, respectively. Mutations are marked by blue blocks along the sequence reads. Sequence traces were obtained from NextGENe software v.2.3.3 (Soft Genetics).

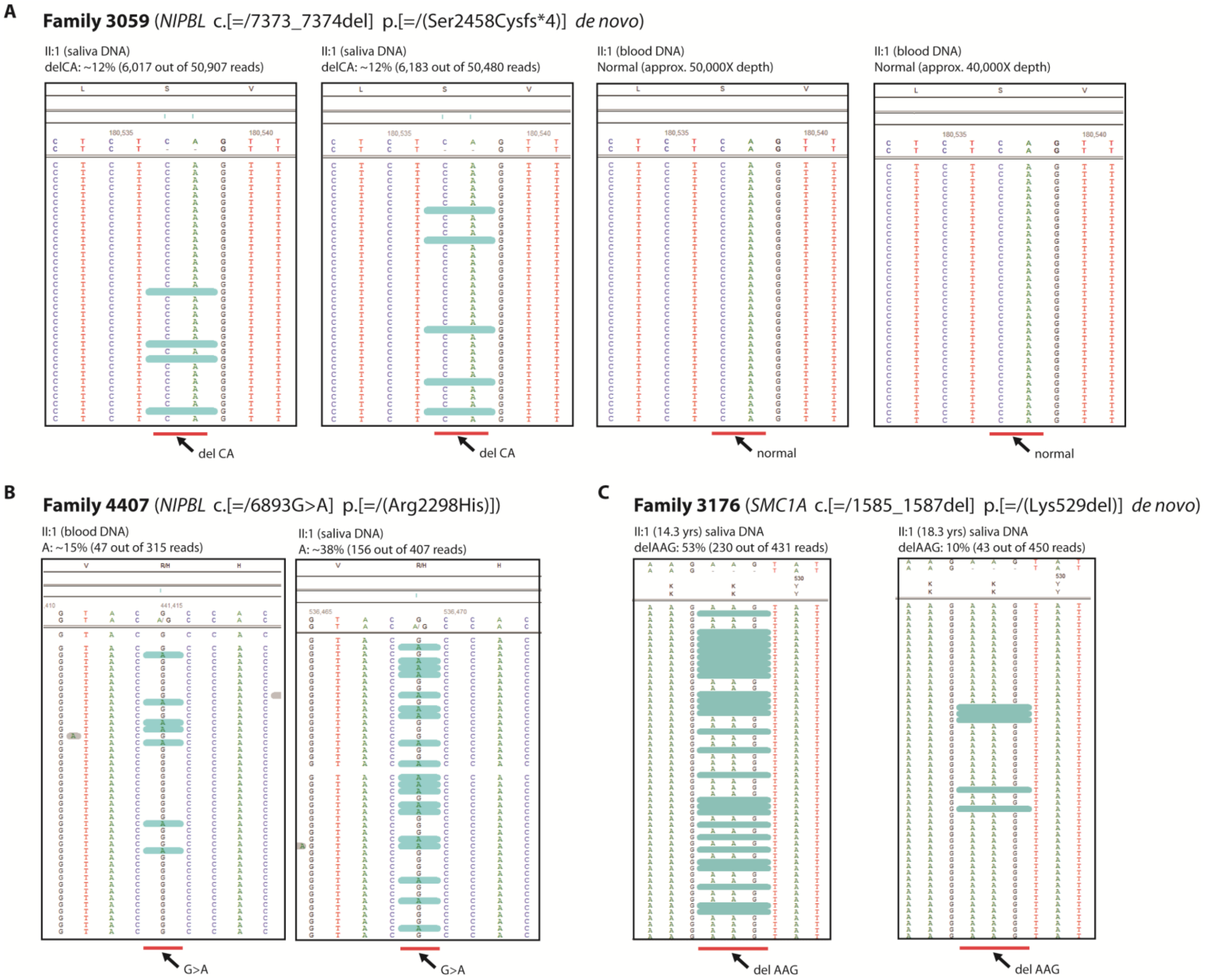
A
Family 3059 (NIPBL c.[=/7373_7374del] p.[=/(Ser2458Cysfs*4)] de novo)
II:1 (saliva DNA)
delCA: ~12% (6,017 out of 50,907 reads)
II:1 (saliva DNA)
delCA: ~12% (6,183 out of 50,480 reads)
II:1 (blood DNA)
Normal (approx. 50,000X depth)
II:1 (blood DNA)
Normal (approx. 40,000X depth)
del CA
del CA
normal
normal
B
Family 4407 (NIPBL c.[=/6893G>A] p.[=/(Arg2298His)])
II:1 (blood DNA)
A: ~15% (47 out of 315 reads)
II:1 (saliva DNA)
A: ~38% (156 out of 407 reads)
G>A
G>A
C
Family 3176 (SMC1A c.[=/1585_1587del] p.[=/(Lys529del)] de novo)
II:1 (14.3 yrs) saliva DNA
delAAG: 53% (230 out of 431 reads)
II:1 (18.3 yrs) saliva DNA
delAAG: 10% (43 out of 450 reads)
del AAG
del AAG

Supplementary figure S2. Genome-wide array CGH analysis of mutation-negative cases. (A) *De novo* heterozygous deletion of 1.3Mb is marked along chromosome 1 (red arrow) in data analysed in Pat ID 3076 (arr 1p36.33 (984,137-2,284,140; hg19)). The results show absence of the deletion in both parents (Fam ID 3076). (B) Heterozygous duplication of 520 kb is marked (blue arrow) on array CGH data from chromosome 12 in Pat ID 3040 (arr 12q13.13 (53,582,733-54,102,733; hg19)). Black dots represent the segmented probes along each chromosome. Log_2 ratio (case:control) is shown on the y-axis and the genomic context is represented along the x-axis using the software programme Signal Map (Roche Nimblegen). (C) The duplication of *ESPL1* was confirmed using a TaqMan CNV assay and compared to seven control samples with either known intragenic causative mutations in another gene (C1-C3) or normal *ESPL1* copy number based on whole-exome data (C4-C7). Genomic copy number, calculated as $2^{\Delta\Delta Ct}$, is shown on the y-axis. Analysis was carried out in triplicate using the software Copy Caller v1.0 (Applied Biosystems).

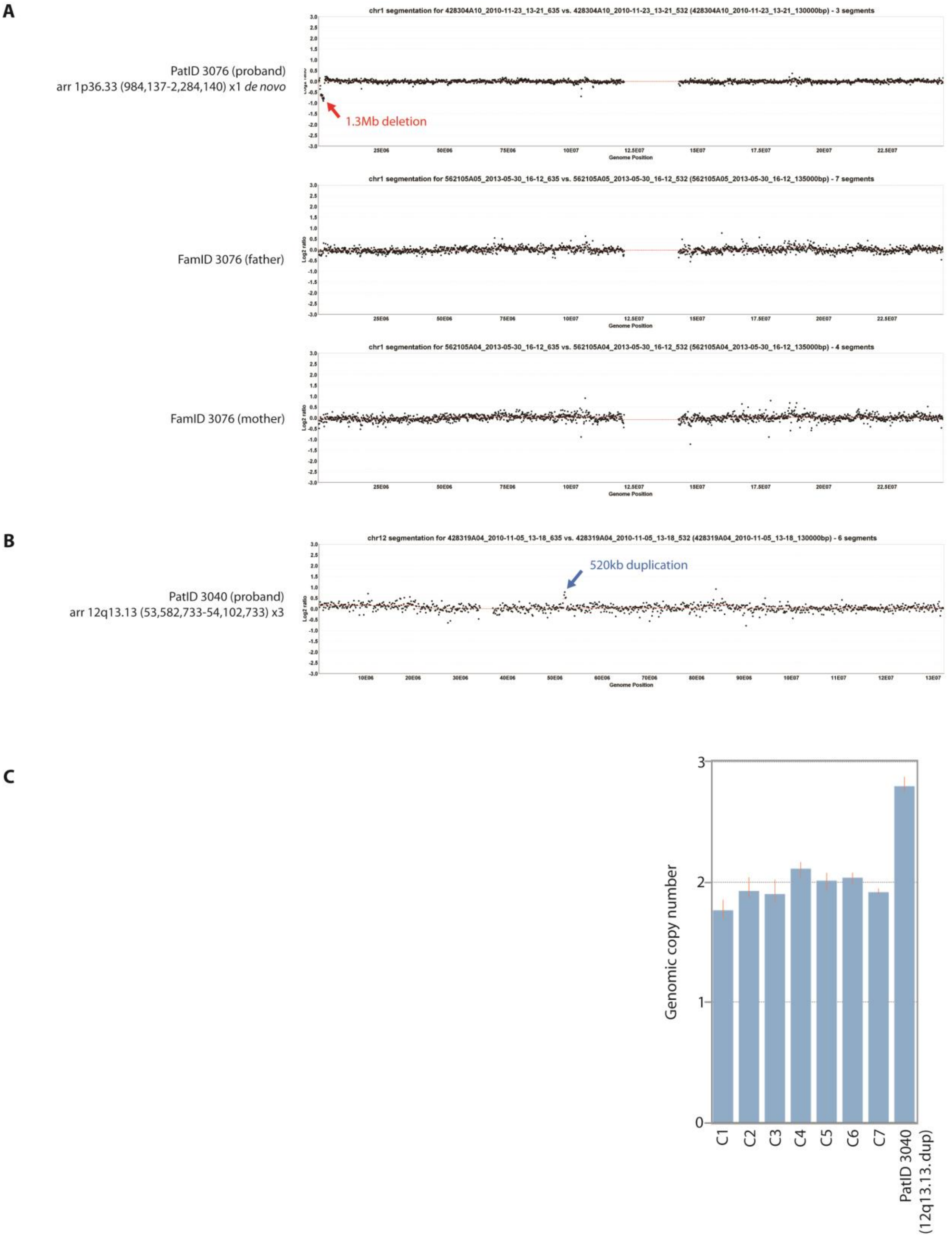
A
PatID 3076 (proband)
arr 1p36.33 (984,137-2,284,140) x1 de novo
chr1 segmentation for 428304A10_2010-11-23_13-21_635 vs. 428304A10_2010-11-23_13-21_532 (428304A10_2010-11-23_13-21_130000bp) - 3 segments
1.3Mb deletion
FamID 3076 (father)
chr1 segmentation for 562105A05_2013-05-30_16-12_635 vs. 562105A05_2013-05-30_16-12_532 (562105A05_2013-05-30_16-12_135000bp) - 7 segments
FamID 3076 (mother)
chr1 segmentation for 562105A04_2013-05-30_16-12_635 vs. 562105A04_2013-05-30_16-12_532 (562105A04_2013-05-30_16-12_135000bp) - 4 segments
Genome Position
B
PatID 3040 (proband)
arr 12q13.13 (53,582,733-54,102,733) x3
chr12 segmentation for 428319A04_2010-11-05_13-18_635 vs. 428319A04_2010-11-05_13-18_532 (428319A04_2010-11-05_13-18_130000bp) - 6 segments
520kb duplication
Genome Position
C
Genomic copy number
C1
C2
C3
C4
C5
C6
C7
PatID 3040 (12q13.13. dup)

Supplementary figure S3. Developmental milestones for individuals with mutations in *NIPBL, SMC1A, SMC3*, *HDAC8, RAD21, ANKRD11*, CNV, and for those without a mutation identified. Ages at which each developmental milestone was reached is shown on the y-axis in months. The number of cases with available growth data in each genotype category is shown at the bottom of each box-plot. The width of each box-plot is proportional to the number of cases in each category. The line in the middle of each box is the median. The bottom and top of each box represent the 25th and 75th centiles, respectively. The whiskers represent the 95th centile (if normally distributed) or the minimum/maximum data points. Outlier values are shown by circles.

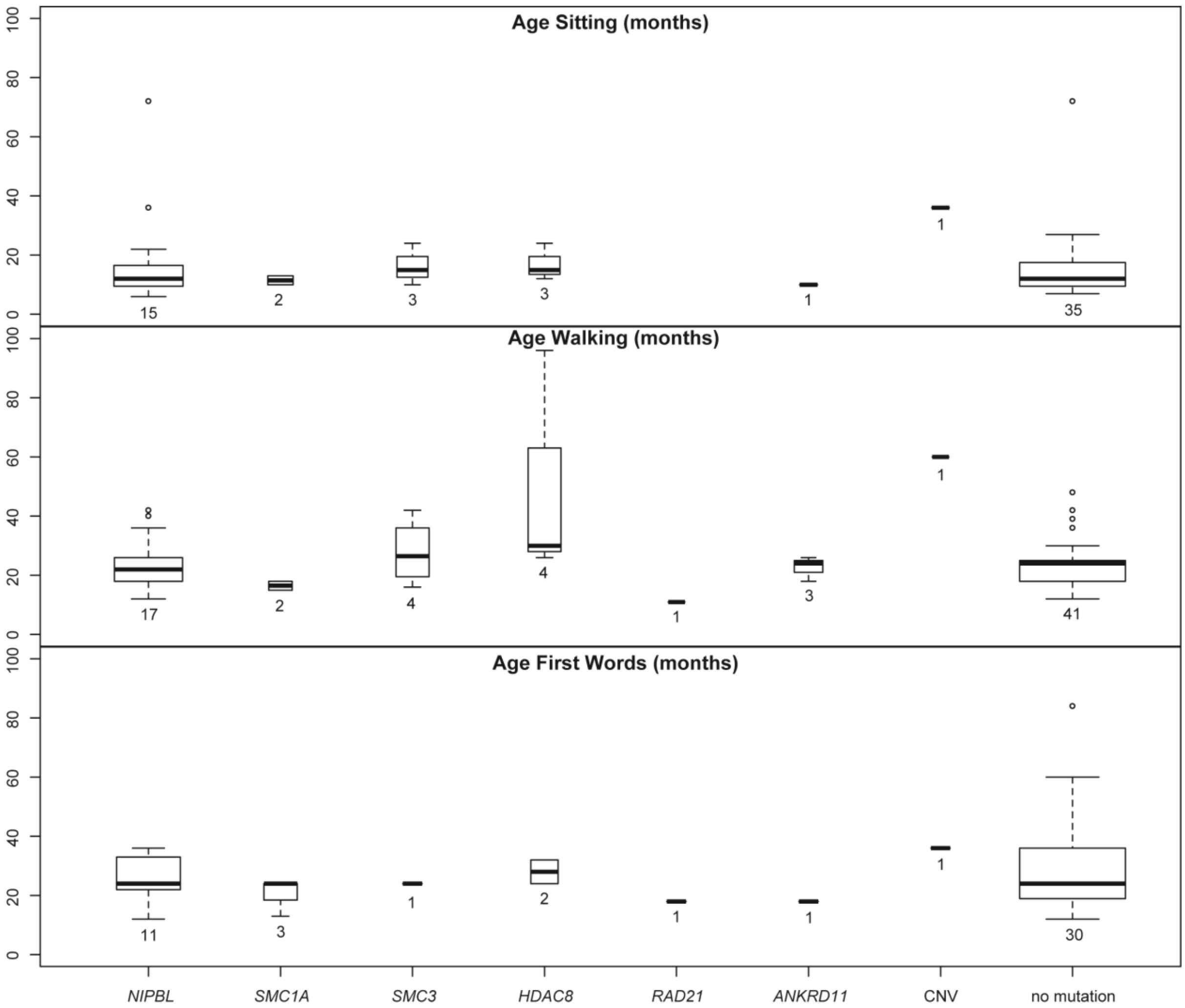

Supplementary figure S4. Box-plots of pre- and post-natal growth for each of the gene categories (*NIPBL*, *SMC1A*, *SMC3*, *HDAC8* and *RAD21*), chromosomal rearrangements (CNV) and those without an identifiable mutation (no_mutation). Box-plots are coloured in red for prenatal growth and purple for postnatal growth. Z scores were calculated using the British Growth Survey data, adjusted for sex and age. The number of cases in each category is shown below the box-plot. BL, birth length; Ht, height; BWt, birth weight; Wt, weight; BOFC, head circumference at birth; OFC, head circumference.

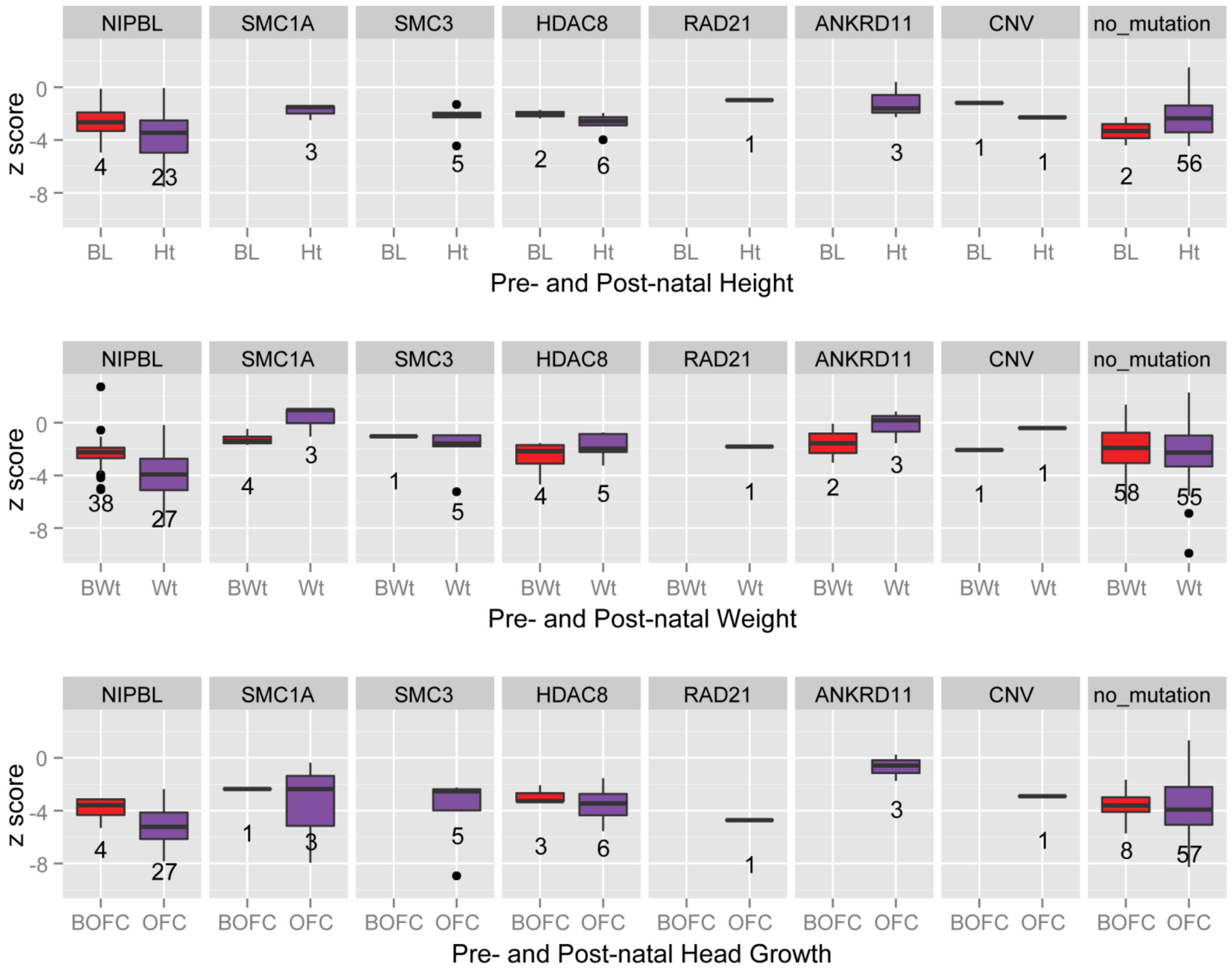
NIPBL
SMC1A
SMC3
HDAC8
RAD21
ANKRD11
CNV
no_mutation
z score
Pre- and Post-natal Height
Pre- and Post-natal Weight
Pre- and Post-natal Head Growth
BL
Ht
BWt
Wt
BOFC
OFC

Supplementary table S1. Type and number of mutations identified in a cohort of 163 affected individuals from whom DNA was available (including mosaic mutations)

Gene and mutation type	Number of mutations (%)	
NIPBL		
	Nonsense	12 (7.4%)
	Frameshift	9 (5.5%)
	Splice site	8 (4.9%)
	Missense*	16 (9.8%)
	Genomic loss/gain	1 (0.6%)†
	Total	46 (28.2%)
SMC1A		
	Nonsense	-
	Frameshift	-
	Splice site	-
	Missense*	5 (3.1%)
	Genomic loss/gain	-
	Total	5 (3.1%)
SMC3		
	Nonsense	1 (0.6%)
	Frameshift	-
	Splice site	-
	Missense*	4 (2.5%)
	Genomic loss/gain	-
	Total	5 (3.1%)
HDAC8		
	Nonsense	-
	Frameshift	-
	Splice site	-
	Missense*	3 (1.8%)§
	Genomic loss/gain	3 (1.8%)§
	Total	6 (3.6%)
RAD21		
	Nonsense	-
	Frameshift	-
	Splice site	1 (0.6%)
	Missense*	-
	Genomic loss/gain	-
	Total	1 (0.6%)

* Includes in-frame insertion/deletions

† Murray *et al*. (2012)

§ Kaiser *et al*. (2014)

Supplementary table S2. Summary of whole-exome sequencing results from five unrelated CdLS and CdLS-like cases

Patient ID	3060	3061	3051	3041	3024
Mean depth	260X	276X	252X	237X	283X
Reads on target	53%	49%	51%	67%	47%
Total number of SNVs (% in dbSNP131)	2346262 (91%)	2710305 (91%)	2434134 (91%)	1116837 (86%)	2432647 (91%)
Total number of indels (% in dbSNP131)	103260 (14%)	140495 (14%)	108481 (14%)	44526 (12%)	102501 (14%)
Filtered* non-synonymous coding	1146	1105	1072	1566	924
Filtered* stop gained	59	50¥	54	73	53
Filtered* frameshift coding	665	679	627	683∞	427
Filtered* essential splice site	14	16	24	33	18

* Not in dbSNP131, 1000 Genomes Pilot projects 1,2,3 or release 2010-08-04, 8 HapMap exomes, or 10 Kabuki syndrome exomes;

¥ contains the mosaic *NIPBL* nonsense mutation, c.[=/1435C>T] p.[=/(Arg479*)];

∞ Contains the *ANKRD11* c.6210_211del p.(Lys2070Asnfs*31) frameshift mutation;

SNV, single nucleotide variant; indel, insertion/deletion

Supplementary table S3. Genotype-phenotype correlations in unrelated cases of CdLS

		**Z score birth length* [95% C.I.] (*N*)**	**Z score birth weight* [95% C.I.] (*N*)**	**Z score birth head circumference* [95% C.I.] (*N*)**	**Z score height* [95% C.I.] (*N*)**	**Z score weight* [95% C.I.] (*N*)**	**Z score head circumference* [95% C.I.] (*N*)**	**Mean Gestalt score† [95% C.I.] (*N*)**	**Mean Severity score [95% C.I.] (*N*)**
NIPBL (all)		-2.59 [-5.72 to 0.55] (4)	-2.32 [-2.76 to -1.88] (38)	-3.88 [-5.53 to -2.23] (4)	-3.68 [-4.45 to -2.91] (23)	-3.91 [-4.67 to -3.15] (27)	-5.14 [-5.74 to -4.53] (27)	7.42 [6.77 to 8.08] (28)	19.63 [16.85 to 22.41] (34)
	nonsense/frameshift	-	-2.43 [-3.31 to -1.54] (17)	-3.07 [NA] (1)	-4.52 [-6.36 to -2.68] (7)	-4.39 [-5.93 to -2.84] (10)	-5.86 [-6.69 to -5.04] (10)	7.71 [6.79 to 8.63] (11)	20.79 [16.54 to 25.03] (16)
	splice site	-4.93 [NA] (1)	-3.21 [-4.14 to -2.27] (6)	-5.30 [NA] (1)	-3.99 [-6.09 to -1.90] (5)	-3.88 [-6.63 to -1.13] (6)	-5.27 [-7.64 to -2.89] (6)	8.39 [7.15 to 9.63] (6)	23.56 [14.26 to 32.85] (7)
	Missense	-1.80 [-5.44 to 1.83] (3)	-1.88 [-2.27 to -1.48] (14)	-3.58 [-9.04 to 1.88] (2)	-3.29 [-4.19 to -2.38] (10)	-3.49 [-4.35 to -2.64] (11)	-4.39 [-5.24 to -3.53] (10)	6.70 [5.27 to 8.13] (10)	15.80 [12.39 to 19.21] (10)
	Genomic loss/gain	-	-1.33 [NA] (1)	-	-0.06 [NA] (1)	-	-4.57 [NA] (1)	5.67 [NA] (1)	12.00 [NA] (1)
SMC1A		-	-1.23 [-2.08 to -0.38] (4)	-2.36 [NA] (1)	-1.79 [-3.28 to -0.31] (3)	0.30 [-2.61 to 3.21] (3)	-3.56 [-13.31 to 6.19] (3)	4.72 [2.37 to 7.08] (3)	13.88 [8.79 to 18.97] (5)
SMC3		-	-1.04 [NA] (1)	-	-2.41 [-3.89 to -0.92] (5)	-2.10 [-4.33 to 0.13] (5)	-4.02 [-7.54 to -0.49] (5)	5.20 [2.13 to 8.27] (5)	10.70 [NA] (1)
HDAC8		-2.04 [-6.11 to 2.03] (2)	-2.65 [-4.94 to -0.35] (4)	-2.87 [-4.62 to -1.12] (3)	-2.71 [-3.46 to -1.96] (6)	-1.82 [-3.09 to -0.53] (5)	-3.52 [-5.01 to -2.02] (6)	5.20 [2.88 to 7.52] (5)	18.37 [14.43 to 22.32] (4)
RAD21		-	-	-	-0.97 [NA] (1)	-1.83 [NA] (1)	-4.71 [NA] (1)	-	-
ANKRD11		-	-1.56 [-20.37 to 17.25] (2)	-	-1.15 [-4.62 to 2.32] (3)	-0.18 [-3.22 to 2.87] (3)	-0.68 [-3.13 to 1.76] (3)	4.78 [2.87 to 6.69] (3)	15.20 [5.03 to 25.36] (2)
Genomic rearrangements		-1.19 [NA] (1)	-2.07 [NA] (1)	-	-2.29 [NA] (1)	-0.41 [NA] (1)	-2.90 [NA] (1)	5.67 [NA] (1)	24.00 [NA] (1)
No mutation		-3.33 [-16.86 to 10.21] (2)	-2.02 [-2.46 to -1.57] (58)	-3.51 [-4.57 to -2.46] (8)	-2.37 [-2.72 to -2.02] (56)	-2.18 [-2.78 to -1.58] (55)	-3.63 [-4.17 to -3.10] (57)	5.63 [5.11 to 6.15] (53)	17.73 [15.84 to 19.63] (48)

* Z scores obtained using the British Growth Survey data; *N*, number of individuals; C.I., confidence interval of the mean; NA, not applicable

Supplementary table S4. Structural anomalies in individuals with an identified mutation.

**System**	**Gene**	**Mutation type**	**Details**
Cardiac	*NIPBL*	Splice	PFO; pulmonary valve stenosis
		Truncating	PFO
		Truncating	pulmonary artery stenosis
		Truncating	ASD; VSD
		Truncating	ASD; VSD; PDA
		Truncating	ASD; VSD
	HDAC8		ASD; VSD
			ASD
	SMC1A		bicuspid aortic valve
Limb reduction defects	*NIPBL*	Splice	phocomelia right arm; micromelia left arm with one digit missing
		Splice	
		Truncating	absent ulna; left hand has thumb and index finger only
		Truncating	right single digit; left four digits; single bone in right forearm
		Truncating	
		Truncating	
		Missense	bilateral thumb hypoplasia
Gastrointestinal	*NIPBL*	Missense	tracheoesophageal fistula
		Splice	pyloric stenosis
		Truncating	pyloric stenosis
		Truncating	malrotation
	SMC3		volvulus on two occasions
Ocular	*NIPBL*	Truncating	myopia -2 dioptres
		Truncating	cataract

PFO = patent foramen ovale, ASD = atrial septal defect, VSD = ventricular septal defect, PDA = patent ductus arteriosus.
